# Supplementary material for: Development and In Vitro Cytotoxicity Evaluation of Individual and Combined Injectable Solutions of Curcumin and Resveratrol Against Lung Cancer Cells
Source: Antioxidants (Basel). 2025 Aug 11;14(8):983. doi: 10.3390/antiox14080983 (PMC12383060; doi:10.3390/antiox14080983)
Supplement: Supplementary file 1 [file antioxidants-14-00983-s001.zip › antioxidants-3791100-supplementary.pdf]

Supplementary Table 1. Experimental design matrix showing the factors, levels and response variables considered in the development of injectable CUR formulation.

Formulation	Coded level			Uncoded level			Response variables	
	CUR	PEG 400	Ethanol	CUR (%)	PEG 400 (%)	Ethanol (%)	Concentration of CUR (mg/mL)	pH
1	-1	-1	-1	0.2	0	10	0.02	5.74
2	-1	1	-1	0.2	60	10	1.96	6.85
3	-1	-1	1	0.2	0	30	0.04	6.10
4	-1	1	1	0.2	60	30	1.94	7.00
5	1	-1	-1	0.3	0	10	0.02	5.78
6	1	1	-1	0.3	60	10	2.93	6.92
7	1	-1	1	0.3	0	30	0.08	6.33
8	1	1	1	0.3	60	30	2.95	7.40

Supplementary Table 2. Experimental design matrix showing the factors, levels and response variables considered in the development of injectable RES formulation.

Formulation	Coded level			Uncoded level			Response variables	
	PG	PEG 400	Ethanol	PG (%)	PEG 400 (%)	Ethanol (%)	Concentration of RES (mg/mL)	pH
1	1	1	-1	60	60	10	89	6
2	-1	1	-1	0	60	10	100.8	6.6
3	-1	0	0	0	30	20	80.1	6.6
4	-1	1	1	0	60	30	93.5	6.7
5	-1	1	0	0	60	20	102.2	6.7
6	-1	-1	0	0	0	20	37.5	6.5
7	-1	-1	1	0	0	30	99.8	6
8	-1	-1	-1	0	0	10	10.4	6.4
9	-1	0	-1	0	30	10	95.2	5.8
10	-1	0	1	0	30	30	92.3	5.4
11	0	-1	-1	30	0	10	93.1	6.3

12	0	-1	1	30	0	30	97.8	6.7
13	0	1	0	30	60	20	98.3	6.4
14	0	0	0	30	30	20	97	6.5
15	0	0	-1	30	30	10	99.1	6.7
16	0	1	1	30	60	30	92.5	6.8
17	0	1	-1	30	60	10	93.1	6.5
18	0	0	1	30	30	30	98.8	6.6
19	0	-1	0	30	0	20	89.8	6.9
20	1	0	1	60	30	30	95.7	6.5
21	1	1	1	60	60	30	98.2	6.7
22	1	0	-1	60	30	10	93.6	7.4
23	1	-1	1	60	0	30	102.4	6.4
24	1	-1	-1	60	0	10	90.7	6.8
25	1	0	0	60	30	20	92.8	6.4
26	1	-1	0	60	0	20	89.6	6.8
27	1	1	0	60	60	20	95.4	6.6
